# Environmental Enrichment Modified Epigenetic Mechanisms in SAMP8 Mouse Hippocampus by Reducing Oxidative Stress and Inflammaging and Achieving Neuroprotection

**DOI:** 10.3389/fnagi.2016.00241

**Published:** 2016-10-18

**Authors:** Christian Griñan-Ferré, Dolors Puigoriol-Illamola, Verónica Palomera-Ávalos, David Pérez-Cáceres, Júlia Companys-Alemany, Antonio Camins, Daniel Ortuño-Sahagún, M. Teresa Rodrigo, Mercè Pallàs

**Affiliations:** ^1^Department of Pharmacology, Toxicology and Therapeutic Chemistry (Pharmacology Section) and Institute of Neuroscience, University of BarcelonaBarcelona, Spain; ^2^Animal Experimentation Unit, Faculty of Pharmacy, University of BarcelonaBarcelona, Spain; ^3^Instituto de Investigación en Ciencias Biomédicas, Centro Universitario de Ciencias de la Salud, Universidad de GuadalajaraGuadalajara, Mexico

**Keywords:** neurodegeneration, environmental enrichment, oxidative stress, epigenetics, inflammation

## Abstract

With the increase in life expectancy, aging and age-related cognitive impairments are becoming one of the most important issues for human health. At the same time, it has been shown that epigenetic mechanisms are emerging as universally important factors in life expectancy. The Senescence Accelerated Mouse P8 (SAMP8) strain exhibits age-related deterioration evidenced in learning and memory abilities and is a useful model of neurodegenerative disease. In SAMP8, Environmental Enrichment (EE) increased DNA-methylation levels (5-mC) and reduced hydroxymethylation levels (5-hmC), as well as increased histone H3 and H4 acetylation levels. Likewise, we found changes in the hippocampal gene expression of some chromatin-modifying enzyme genes, such as *Dnmt3b. Hdac1. Hdac2. Sirt2*, and *Sirt6.* Subsequently, we assessed the effects of EE on neuroprotection-related transcription factors, such as the Nuclear regulatory factor 2 (Nrf2)–Antioxidant Response Element pathway and Nuclear Factor kappa Beta (NF-κB), which play critical roles in inflammation. We found that EE produces an increased expression of antioxidant genes, such as *Hmox1. Aox1*, and *Cox2*, and reduced the expression of inflammatory genes such as *IL-6* and *Cxcl10*, all of this within the epigenetic context modified by EE. In conclusion, EE prevents epigenetic changes that promote or drive oxidative stress and inflammaging.

## Introduction

With the increase in the average life expectancy of the population worldwide, cognitive frailty is emerging as one of the most important issues of human health, with a rising percentage of the population affected by cognitive decline and dementia. However, it remains impossible to delineate the precise moment at which the brain begins to age (Fjell et al., 2014).

Alzheimer’s Disease (AD) is the most prevalent dementia and its progression is influenced by both genetic and environmental factors (Grupe et al., 2007; Avramopoulos, 2009; Harold et al., 2009). These gene–environment interactions may influence and trigger pathogenic pathways that determine the severity and progression of several degenerative diseases (Mastroeni et al., 2009; Bradley-Whitman and Lovell, 2013; Millan, 2014; Griñán-Ferré et al., 2015). In addition, a growing body of evidence demonstrated that the effects of environmental factors are mainly orchestrated by epigenetic mechanisms. In AD, only a small percentage of the cases (<5%) are consistent with inheritance. Even in the familial forms of AD, penetrance is not complete and is also highly variable between individuals with the same genetic background (Mastroeni et al., 2009, 2011). Consequently, the effect of environmental factors is of remarkable importance. In this respect, epigenetic changes, or other forms of gene-environment interaction, could be involved in determining the onset of AD and also aging, which is the most important risk factor for development of the pathology (López-Otín et al., 2013; Spiegel et al., 2014).

In particular, lines of evidence indicate that the methylation and hydroxymethylation state of the DNA plays an important role in controlling gene expression, and also learning and memory, including memory formation and maintenance of learning and memory decline during aging (Levenson et al., 2004; Miller and Sweatt, 2007; Liu et al., 2009; Day and Sweatt, 2010; Mikaelsson and Miller, 2011; Morris et al., 2014).

Similarly, epigenetic mechanisms, i.e., alterations of histone proteins and DNA, are essential for hippocampal synaptic plasticity and cognitive function disease (López-Otín et al., 2013). Additionally, recent works (Holliday, 2006; Fischer et al., 2007; Gräff and Tsai, 2013) demonstrate that chromatin regulation, and in particular histone acetylation, could be important for modulating the cognitive outcome of several neurodegenerative disorders. Moreover, exposure to an EE has been shown to have a protective effect in mouse models by slowing disease progression and reducing AD-like cognitive impairment (Barak et al., 2013).

Although the mechanisms by which EE preserves cognition remain unknown, our work team recently demonstrated that EE is an experimental strategy to alleviate cognitive decline and neuroprotection in Senescence Accelerated Mice P8 (SAMP8) (Griñán-Ferré et al., 2015). Moreover, changes in modulators of epigenetic machinery, such as LSD1 or CoREST, were found changed after EE in this strain and were accompanied by cognitive feature improvements (Griñán-Ferré et al., 2015). These results correlated with the activation of endogenous neuroprotective pathways, thus implicating these in the beneficial effects of EE on cognition.

Many studies have demonstrated that EE changes promoter DNA methylation states while changing the expression of DNA MethylTransferases (DNMTs) (Christensen et al., 2009; Marsit et al., 2009; Madrigano et al., 2011; Barrès et al., 2012). Therefore, gene-environment interactions, mediated at the epigenetic level, may be an intermediary step in providing an appropriate organism response to changes in the environment (Irier et al., 2014).

There is evidence that EE attenuated Oxidative Stress (OS) through elevated Nrf2 levels in hippocampi (Snow et al., 2015). In addition, EE elicits anti-inflammatory effects through interaction with several immune signaling pathways, including Interleukin 6 (IL-6). Diminution in the proinflammatory process after EE might be due by changes in NF-κB translocation to the nucleus, reducing chemokines (Costa et al., 2007; Jurgens and Johnson, 2012; Williamson et al., 2012).

The goal of the present work was to delve deep into the epigenetic mechanisms influenced by EE that rendered the previously described beneficial effects by preventing cognitive impairment and neuronal dysfunction. We focus on antioxidant and inflammaging processes modulated by EE exposure in SAMP8.

## Materials and Methods

### Animals and Enriched Environmental Experimental Design

Male SAMP8 mice (*n* = 20) were used with free access to food and water, under standard temperature conditions (22 ± 2°C) and 12-h:12-h light-dark cycles (300 lux/0 lux).

The animals were maintained until day 21 with their mothers, and afterward were separated into cages at up to 3 months-of-age. Ten SAMP8 were employed for the Environmental Enrichment (EE) group (SAMP8 EE), and 10 were maintained under standard conditions as Control mice (SAMP8 Ct).

In the present study, we utilized the novel objects paradigm. Therefore, plastic tubes (20 cm long and 2.5 cm in diameter) were placed in EE cardboard-house cages, in addition to plastic dolls or toys, which were added, extracted, or changed each week.

Mice were treated according to European Community Council Directive 86/609/EEC and the procedures established by the Department d’Agricultura, Ramaderia i Pesca of the Generalitat de Catalunya, Spain. Every effort was made to minimize animal suffering and to reduce the number of animals.

### Behavioral and Cognitive Experiments

#### Novel Object Recognition Test (NORT)

The protocol employed was a modification of Ennaceur and Delacour (1988) and Ennaceur and Meliani (1992). In brief, mice were placed in a 90°-, two-arm, 25-cm-long, 20-cm-high, 5-cm-wide black maze. The walls could be removed for easy cleaning. Light intensity in mid-field was 30 lux. The objects to be discriminated were made of plastic and were chosen not to frighten the mice, and objects with parts that could be bitten were avoided. Before performing the test, the mice were individually habituated to the apparatus for 10 min during 3 days. On day 4, the animals were submitted to a 10-min acquisition trial (first trial), during which they were placed in the maze in the presence of two identical, novel objects (A+A or B+B) at the end of each arm. A 10-min retention trial (second trial) was carried out 2 h later. During this second trial, objects A and B were placed in the maze, and the behavior of the mice was recorded with a camera. Time that mice explored the New object (TN) and Time that mice explored the Old object (TO) were measured. A Discrimination Index (DI) was defined as (TN-TO)/(TN+TO). In order to avoid object preference biases, objects A and B were counterbalanced so that one half of the animals in each experimental group were first exposed to object A and then to object B, whereas the other half first saw object B and then object A. The maze and the objects were cleaned with 96° ethanol after each test in order to eliminate olfactory cues.

#### Morris Water Maze Test

An open circular pool (100 cm in diameter, 50 cm in height) was filled halfway with water (Morris, 1981) and the temperature was maintained at 22°C ± 1. Two principal perpendicular axes were defined; thus, the water surface was divided into four quadrants (NE, SE, SW, and NW) and five starting points were set (NE, E, SE, S, and SW). Four visual clues were placed on the walls of the tank (N, E, S, and W). Non-toxic, white latex paint was added to make the water opaque, and a white escape platform was submerged 1 cm below the water level (approximately in the middle of one of the quadrants).

The animals’ swimming paths were recorded by a video camera mounted above the center of the pool, and data were analyzed with SMART version 3.0 software. The learning phase consisted of 6 days of trials for each mouse. The animals were submitted to five trials each day starting from the positions set (in random order) and without a resting phase between each trial and the subsequent one. At each trial, the mouse was placed gently into the water, facing the wall of the pool, and allowed to swim for 60 s. If not able to locate the platform in this period of time, the mouse was guided to the platform by the investigator. Animals were left on the platform each time for 30 s in order to allow for spatial orientation. A memory test was performed at the end of the learning days, in which the platform was removed and the time spent and the distance swum by each mouse in each quadrant was measured.

### Immunodetection Experiments

#### Brain Processing and Subcellular Fractionation

After the behavioral test, the animals were killed by cervical dislocation. Afterward, the brains were immediately removed and the hippocampi were then isolated, frozen on powdered dry ice, and maintained at -80°C until protein extraction.

For subcellular fractionation, 150 μL of buffer A (10 mM HEPES pH 7.9, 10 mM KCl, 0.1 mM EDTA pH 8, 0.1 mM EGTA pH 8, 1 mM DTT, 1 mM PMSF, protease inhibitors) were added to each sample and incubated on ice for 15 min. After this time, the samples were homogenized with a tissue homogenizer, 12.5 μL Igepal 1% were added, and the Eppendorf was vortexed for 15 s. Following 30 s of full-speed centrifugation at 4°C, supernatants were collected (cytoplasmic fraction); 80 μL of buffer C (20 mM HEPES pH 7.9, 0,4M NaCl, 1 mM EDTA pH 8, 0.1 mM EGTA pH 8, 20% Glycerol 1 mM DTT, 1 mM PMSF, protease inhibitors) was added to each pellet and incubated under agitation at 4°C for 15 min. Subsequently, samples were centrifuged for 10 min at full speed at 4°C. Supernatants were collected (nuclear fraction) and 40 μL of buffer A+HCl (buffer A with 0.2 N HCl) was added to the pellet. After a 30-min incubation on ice, samples were centrifuged, again at full speed, at 4°C for 10 min and the supernatants were collected (the histone fraction).

#### Western Blotting

For the Western blot (wb) experiment, aliquots of homogenized hippocampus, 15 μg protein for nuclear and cytoplasmic fractions, and 5 μg for the histone fraction were used. The protein samples were separated by SDS–PAGE (8–18%) and transferred onto PVDF membranes (Millipore). The membranes were blocked in 5% non-fat milk in TBS containing 0.1% Tween 20 (TBS-T) for 1 h at room temperature, followed by an overnight incubation at 4°C with the primary antibodies listed in **Table 1**. Membranes were then washed and incubated with secondary antibodies for 1 h at room temperature. Immunoreactive protein was viewed with a chemiluminescence-based detection kit, following the manufacturer’s protocol (ECL Kit; Millipore) and digital images were acquired using a ChemiDoc XRS+ System (BioRad). Semi-quantitative analyses were carried out utilizing Image Lab software (BioRad) and results were expressed in Arbitrary Units (AU). Protein loading was routinely monitored by phenol red staining of the membrane or by immunodetection of GADPH.

**Table 1:** Antibodies used in Western blot studies.

Antibody	Host	Source/Catalog	WB dilution
p65	Rabbit	Cell signaling/D14E12	1:1000
NRF2	Rabbit	Cell signaling/DlZ9C	1:1000
Acetyl Histone H3	Rabbit	Millipore/06-599	1:1000
Acetyl Histone H4	Sheep	R&D systems/AF5215	1:1000
GAPDH	Mouse	Millipore/MAB374	1:2000
Histone H3	Rabbit	Santa Cruz Biotech/sc-10809	1:500
Histone H4	Mouse	Cell signaling/L64Cl	1:1000
TBP	Mouse	Abcam/51841	1:1000
Donkey-anti-goat HRP conjugated		Santa Cruz Biotech/ sc-2020	1:3000
Goat-anti-mouse HRP conjugated		Biorad/# 170-5047	1:2000
Rabbit-anti-sheep HRP conjugated		Abcam/ab97130	1:2000
Goat-anti-rabbit HRP conjugated		Cell signaling/tt 7074	1:2000


#### Global DNA Methylation and Hydroxymethylation Quantification

For global DNA, methylation and hydroxymethylation was performed according to the manufacturer’s instructions, first using the FitAmpTM Blood and Cultured Cell DNA Extraction Kit, which is designed for rapid isolation of pure genomic DNA from a small amount of blood or mammalian cells. The second part was performed employing the Methylflash Methylated DNA Quantification Kit (Epigentek, Farmingdale, NY, USA) and the Methylflash Hydroxymethylated DNA Quantification Kit. Briefly, methylated DNA and hydroxymethymethylated DNA were detected using capture and detection antibodies to 5-mC and 5-hmC and then quantified colorimetrically by reading absorbance at 450 nm using the Microplate Photometer. The absolute amount of methylated or hydroxymethylated DNA (proportional to the Optical Density [OD] intensity) was measured and was quantified using a standard curve plotting the OD values vs. five serial dilutions of control methylated DNA (0.5–10 ng).

#### RNA Extraction and Gene Expression Determination

Total RNA isolation was carried out by means of Trizol reagent following the manufacturer’s instructions. RNA content in the samples was measured at 260 nm, and sample purity was determined by the A260/280 ratio in a NanoDrop^TM^ ND-1000 (Thermo Scientific). Samples were also tested in an Agilent 2100B Bioanalyzer (Agilent Technologies) to determine the RNA integrity number. Reverse Transcription-Polymerase Chain Reaction (RT-PCR) was performed as follows: 2 μg of messenger RNA (mRNA) was reverse-transcribed using the High Capacity complementary DNA (cDNA) Reverse Transcription Kit (Applied Biosystems). Real-time quantitative PCR (qPCR) was employed to quantify the mRNA expression of a set of chromatin- modifying enzyme genes, including *Dnmt1. Dnmt3a. Dnmt3b*, methylcytosine dioxygenase TET1 (*Tet1*), methylcytosine dioxygenase TET2 (*Tet2*), Sirtuin 1 (*Sirt1*), Sirtuin 2 (*Sirt2*), Sirtuin 6 (*Sirt6*), Histone deacetylase 1 (*Hdac1*), Histone deacetylase 2 (*Hdac2*), OS genes Heme oxygenase (decycling) 1 (*Hmox1*), Aldehyde oxidase 1 (*Aox1*), Aldehyde dehydrogenase 2 (*Aldh2*), Cyclooxygenase 2 (*Cox2*), inflammatory genes Interleukin 6 (*IL-6*), C-X-C motif chemokine 10 (*Cxcl10*), and Tumor necrosis factor alpha (*Tnf-α*). Normalization of expression levels was performed with actin for SYBER Green and TATA-binding protein (*Tbp*) for TaqMan. The primers were those listed in **Table 2**.

**Table 2:** Primers and probes used in qPCR studies.

Target	Product size (bp)	Forward primer (5′-3′)	Reverse primer (5′-3′)
SYBR Green primers			
Dnmt1	85	ACCTGGAGAGCAGAAATGGC	TGAAAGGGTGTCACTGTCCG
Dnmt3b	142	TGCCAGACCTTGGAAACCTC	GCTGGCACCCTCTTCTTCAT
Tet1	188	CTGCCAACTACCCCAAACTCA	TCGGGGTTTTGTCTTCCGTT
Tet2	113	CCATCATGTTGTGGGACGGA	ATTCTGAGAACAGCGACGGT
Hdac1	150	TCACCGAATCCGCATGACTC	TCTGGGCGAATAGAACGCAG
Hdac2	280	CTATCCCGCTCTGTGCCCT	GAGGCTTCATGGGATGACCC
Sirt1	229	AACACACACACAAAATCCAGCA	TGCAACCTGCTCCAAGGTAT
Sirt2	248	TGCAGGAGGCTCAGGATTC	GTCACTCCTTCGAGGGTCAG
Sirt6	150	GTCTCACTGTGTCCCTTGTCC	GCGGGTGTGATTGGTAGAGA
Aox1	286	CATAGGCGGCCAGGAACATT	TCCTCGTTCCAGAATGCAGC
Cox2	126	TGACCCCCAAGGCTCAAATA	CCCAGGTCCTCGCTTATGATC
IL-6	189	ATCCAGTTGCCTTCTTGGGACTGA	TAAGCCTCCGACTTGTGAAGTGGT
Cxcl10	72	GGCTAGTCCTAATTGCCCTTGG	TTGTCTCAGGACCATGGCTTG
Actin	190	CAACGAGCGGTTCCGAT	GCCACAGGTTCCATACCCA

**Target**		**Product size (bp)**	**Reference**

Taqman probes			
Dnmt3a		58	Mm00432881 m1
Hmox1		69	Mm00516005_m1
Tbp		93	Mm00446971_m1


For SYBER Green, real-time PCR was performed in the Step One Plus Detection System (Applied Biosystems) employing the SYBR Green PCR Master Mix (Applied Biosystems). Each reaction mixture contained 7.5 μL of cDNA, whose concentration was 2 μg, 0.75 uL of each primer (whose concentration was 100 nM), and 7.5 μL of SYBR Green PCR Master Mix (2X), and for TaqMan gene expression assays (Applied Biosystems), for each 20 μL of TaqMan reaction, 9 μL cDNA (18 ng) was mixed with 1 μL 20X probe of TaqMan Gene Expression Assays and 10 μL of 2X TaqMan Universal PCR Master Mix.

Data were analyzed utilizing the comparative Cycle threshold (Ct) method (ΔΔCt), where the actin transcript level was used to normalize differences in sample loading and preparation. Each sample (*n* = 4-5) was analyzed in triplicate, and results represented the *n*-fold difference of transcript levels among different samples.

#### Data Analysis

Data are expressed as the mean ± Standard Error of the Mean (SEM) from at least 5–6 samples. Data analysis was conducted using GraphPad Prism version 6 statistical software. Means were compared with the two-tailed, unpaired Student *t-*test. Statistical significance was considered when *p-*values were <0.05.

## Results

### EE Mitigates Cognitive Decline in Male SAMP8 Mice

**FIGURE 1:**
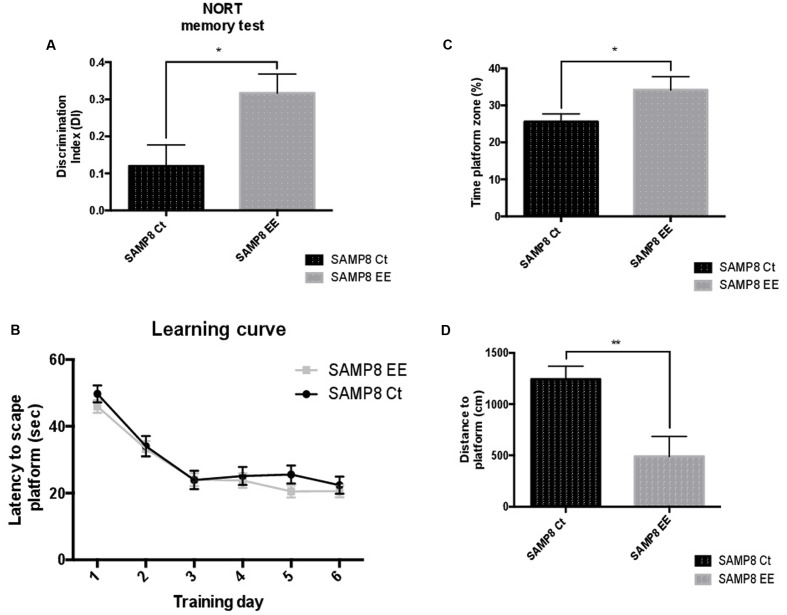
**Results of the Discrimination Index (DI) of the Novel Object Recognition Test (NORT) (**A)**.** Learning curves during Morris Water Maze (MWM) trials **(B)**. Percentage of time spent in platform zone during 60-sec probe trial of the MWM test **(C)**. Distance to platform **(D)**. Values are mean ± Standard Error of the Mean (SEM); *n* = 10. Statistics: *^∗^p* < 0.05; *^∗∗^p* < 0.01; ^∗∗∗^*p* < 0.001, and ^∗∗∗∗^*p* < 0.0001, compared with SAMP8 Ct.

The Novel Object Recognition Test (NORT) demonstrated significant memory amelioration in SAMP8 EE mice in reference to SAMP8 Controls (Ct) (**Figure 1A**). Improvement in cognition was also demonstrated by the results obtained in the Morris Water Maze (MWM) test (**Figure 1B**). Moreover, time spent on platform quadrant was longer (**Figure 1C**); in contrast, distance swum to platform was significantly lesser in SAMP8 EE than in SAMP8 Ct (**Figure 1D**). These results corroborate that previously obtained, but with higher number of animals (Griñán-Ferré et al., 2015).

### Hippocampal Global Changes in DNA Methylation and Hydroxymethylation and Its Machinery after EE

**FIGURE 2:**
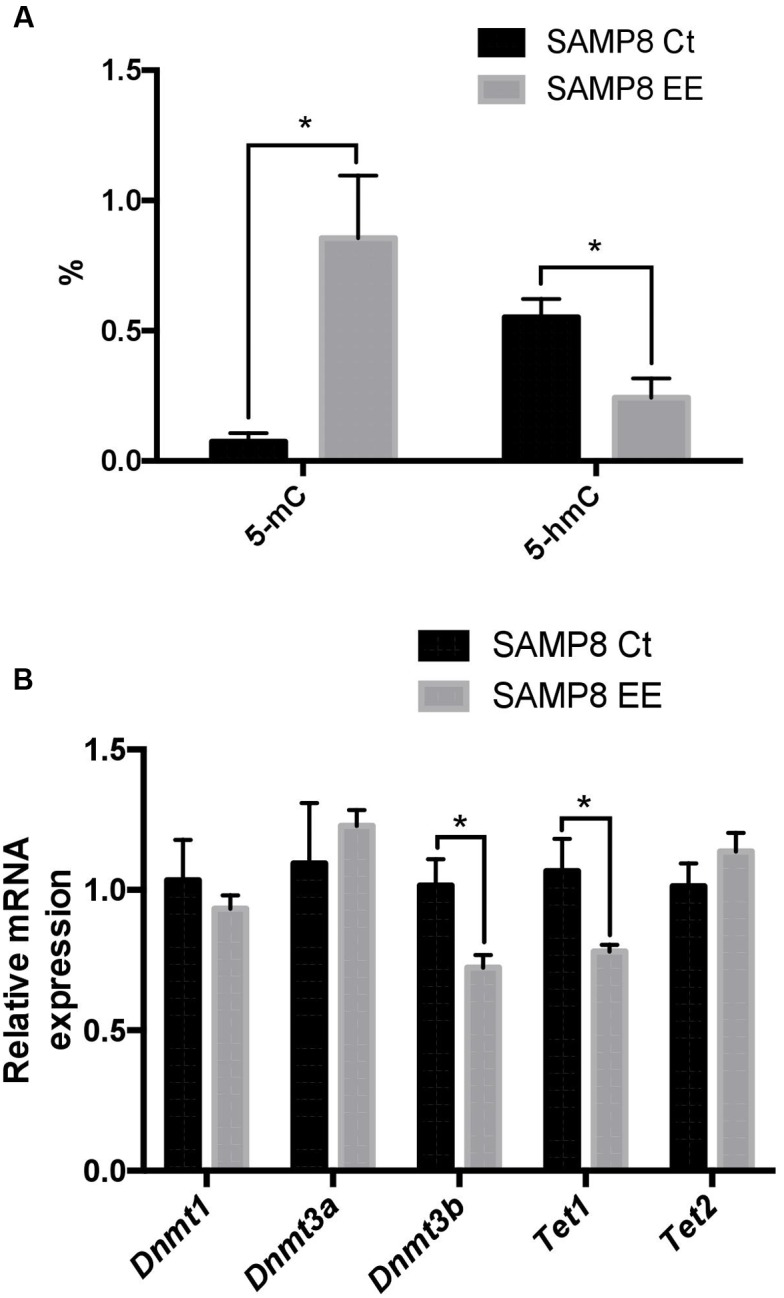
**Global 5-methylated cytosine and 5-hydroxymethylated cytosine levels **(A)**.** Gene expression for *Dnmt1. Dnmt3a. Dnmt3b. Tet1*, and *Tet2* **(B)**. Gene expression levels were determined by real-time PCR. Presented here are Mean ± Standard Values are means (Standard Error of the Mean [SEM]) of the five independent experiments performed in triplicate. ^∗^*p* < 0.05; ^∗∗^*p* < 0.01; ^∗∗∗^*p* < 0.001, and ^∗∗∗∗^*p* < 0.0001 compared with SAMP8 Ct.

To address the question of whether or not EE could affect DNA methylation or hydroxymethylation, we determined 5-methylcytosine levels and found a significant increase in SAMP8 EE in comparison with SAMP8 Ct (**Figure 2A**). In a parallel manner, 5-hmC levels were reduced in SAMP8 EE. When gene expression in DNMTs was studied, only significant decreases in *Dnmt3b* were found (**Figure 2B**). Moreover, Ten-eleven Translocation Methylcytosine Dioxygenase 1 (*Tet1*), but not *Tet2*, increased in SAMP8 EE mice (**Figure 2B**).

### Hippocampal Global Changes in Histone Acetylation Levels and Its Machinery after EE

**FIGURE 3:**
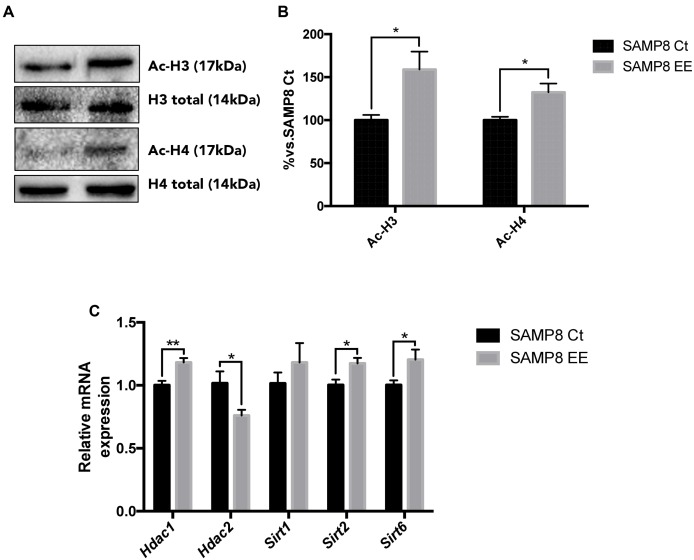
**Representative Western blot (wb) for acetylated and total H3 and H4 protein levels **(A)** and quantification after Enhanced Environment (EE) intervention **(B)**.** Deacetylase gene expression related with memory for *Hdac1. Hdac2. Sirt1. Sirt2*, and *Sirt6* **(C)**. Values in bar graphs are adjusted to 100% for protein levels of Control SAMP8 (SAMP8 Ct). Gene expression levels were determined by real-time PCR. Mean ± Standard Error of the Mean (SEM) from five independent experiments performed in duplicate are represented. ^∗^*p* < 0.05 vs. Control SAMP8 (SAMP8 Ct).

The main Histone DeACetylase (HDAC) members of families I and III were studied. HDAC 1 and 2 (representative of the HDAC class I family) exhibited a contrasting profile, with increased gene expression in *Hdac1* and a decrease in *Hdac2. Sirt2* and *Sirt6*, members of HDAC family III, are increased after EE in SAMP8 (**Figure 3C**). Protein Acetylated H4 was significantly increased in SAMP8 EE, whereas Acetylated H3 showed increased levels that did not reach significance (**Figures 3A,B**), demonstrating that higher levels of *Hdac2* were determining in histone acetylation levels under the EE strategy in SAMP8.

### Molecular Changes in Nrf2 and NF-κB Pathways Induced by EE

**FIGURE 4:**
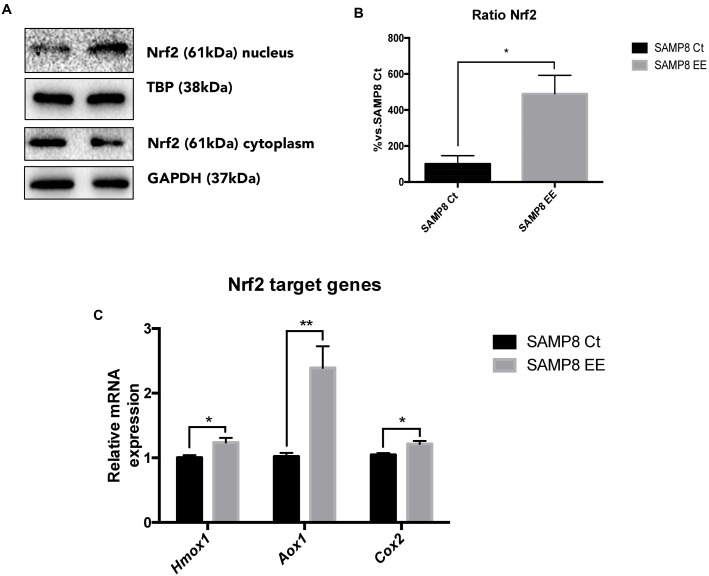
**Representative wb for Nuclear regulatory factor 2 (Nrf2) in cytoplasm and nucleus protein levels **(A)** and ration quantification **(B)**.** Nrf2 target-gene expression *Hmox1. Aox1*, and *Cox2* **(C)**. Values in bar graphs are adjusted to 100% for protein levels of Control SAMP8 (SAMP8 Ct). For gene expression, levels were determined by real-time PCR. Mean ± Standard Error of the Mean (SEM) from five independent experiments performed in duplicate are represented. ^∗^*p* < 0.05 vs. Control SAMP8 (SAMP8 Ct).

To address the question of whether EE increase in antioxidant enzymes is achieved by upregulating Nrf2, we studied protein levels and demonstrated that this transcription factor is translocated into the nucleus in the hippocampus of SAMP8 EE (**Figures 4A,B**). In parallel fashion, expression of *Nrf2* target genes was significantly increased in these animals. Concretely, *Hmox1. Aox1*, and *Cox2*, genes coding for oxidative machinery proteins, were upregulated in SAMP8 EE compared with SAMP8 Ct (**Figure 4C**).

**FIGURE 5:**
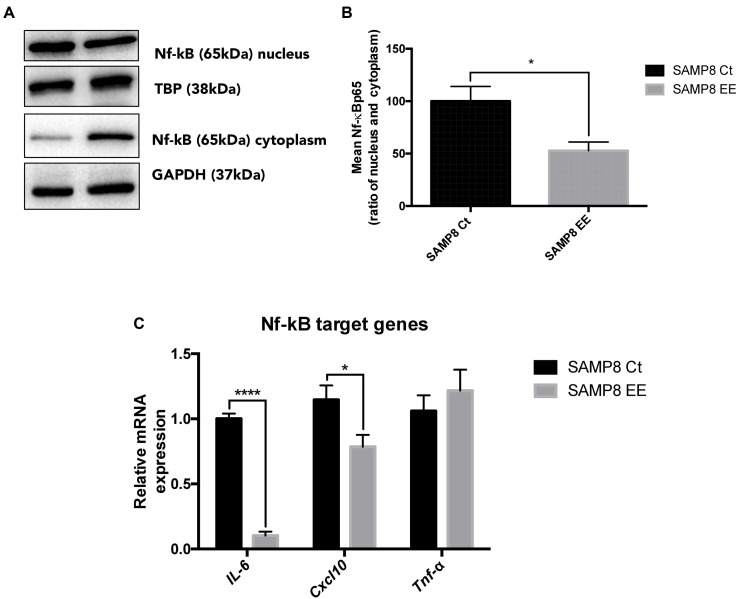
**Representative wb for Nuclear Factor kappa Beta (NF-κB) in cytoplasm and nucleus protein levels **(A)** and ration quantification **(B)**.** NF-κB target-gene expression *IL-6. Cxcl10*, and *Tnf-α* **(C)**. Values in bar graphs are adjusted to 100% for protein levels of Control SAMP8 (SAMP8 Ct). For gene expression, levels were determined by real-time PCR. Mean ± Standard Error of the Mean (SEM) from five independent experiments performed in duplicate are represented. ^∗^*p* < 0.05 vs. Control SAMP8 (SAMP8 Ct).

Previous results from our laboratory demonstrated that *IL-6* expression was lowered by EE in SAMP8. NF-κB is a transcription factor related with inflammatory response and a master commander in the expression of proinflammatory genes. We found that EE reduced translocation to the nucleus (**Figures 5A,B**) and, most importantly, demonstrated that this reduction in nuclear translocation rendered a significantly decrease in *IL-6* and *Cxcl10* gene expression, although no changes were observed in *Tnf-α* (**Figure 5C**).

## Discussion

In the present study, we showed that EE affected epigenetic markers and produced changes in the transcriptional control of oxidative and inflammaging markers in SAMP8. We hypothesize that changing the environment protectively would modulates a large constellation or at least a large subset of cellular phenotypes of aging. SAMP8 comprises a well-enough, but still not fully characterized, model for studying brain aging and neurodegeneration (Takeda, 2009; Pallàs, 2012). Experimental studies on SAMP8 postulated it as a non-transgenic murine model for late-onset AD (Pallàs et al., 2008; Morley et al., 2012b; Pallàs, 2012; Cheng et al., 2014), in addition to being a spontaneous senescence-accelerated mouse strain. These mice exhibited cognitive and emotional disturbances from young ages, probably due to early development of brain pathological hallmarks, such as OS, inflammation, and activation of neuronal death pathways, which mainly affects cerebral cortex and hippocampus (Canudas et al., 2005; Sureda et al., 2006; Cristòfol et al., 2012; Morley et al., 2012a). We have revealed the first, to our knowledge, clue regarding epigenetic modulation in inflammation and oxidative processes in SAMP8 (Alvarez-López et al., 2013) and in a model of familial AD (Griñán-Ferré et al., 2016). Therefore, an adequate epigenetic intervention during early life would be able to have influence on the dysfunctional or harmful molecular mechanisms affected in such a way that this would be able to exert a positive influence (Cosín-Tomás et al., 2014).

Molecular changes in young-age SAMP8 are based on cerebral and cognitive dysfunction (Yuan et al., 2012; Alvarez-López et al., 2013), and these harmful changes were partially reverted in SAMP8 after EE early exposure, including cognitive, psychoemotional, and neurodegenerative hallmarks of AD (Griñán-Ferré et al., 2015). In addition to neuroprotection, EE was able to induce some type of epigenetic modulation in this strain, due to changes in CoREST and LSD1, in both hippocampus and cortex. Therefore, to open the focus on and to probe this epigenetic control, we demonstrated here that the DNA transcriptional control of specific genes indicated its being the initial and key step in the molecular stream developed in SAMP8 by EE, which improves cognition and ameliorates harmful factors gated to neurodegeneration, such as neuronal loss and apoptosis prevention, downregulation of kinase activity, and reduction in OS.

DNA methylation influences hippocampal memory formation, and growing evidence suggests that the regulation of epigenetic processes by EE, stress, and/or hormones can participate in memory function. In the present work and in accordance with the decrease in LSD1 transcription described in Griñán-Ferré et al. (2015), a higher degree of DNA hypermethylation was found in SAMP8 EE hippocampus. Conversely, only DNMT3b was reduced, whereas other DNMT were not modified. Usually, 5-mC decreases transcriptional access to DNA, although the functional effects of this gene silencing depend on the genes that are altered (Alvarez-López et al., 2013). However, recent data suggest a more complex role for 5-mC, which also includes stimulation of gene expression (Weber et al., 2007; Wu and Zhang, 2010). Both, increases and decreases of 5-mC have been described during aging (Richardson, 2003). Furthermore, pathological DNMT activity and aberrant 5-mC formation have been linked with neurodegeneration and apoptotic neuronal death (Chestnut et al., 2011; Hernandez et al., 2011). In these latter studies, neuroprotection was provided by DNMT inhibitors.

5-hmC is another epigenetic marker, catalyzed by the TET family, that appears to be particularly susceptible to developmental and aging-associated modifications (Flax and Soloway, 2011; Ito et al., 2011). According with other studies, diminution in 5-hmC, which paralleled a decrease in the *Tet1* expression level, was found after EE in SAMP8. For instance, Irier et al. (2014) found that aged mice exposed to EE improved learning and memory, reducing 5-hmC-abundant hippocampus. However, in this work, no changes in *Tet1* expression were found, and differences can be explained by different timing in EE and also because of differences in strain and age. In sum, aging-associated 5-hmC alterations appear to be likely participants in the neuroplasticity of the aging brain.

In addition to DNA methylation changes, histone alterations are one of epigenetic mechanisms that are essential for hippocampal synaptic plasticity and cognitive function diseases (Zhao et al., 2012). In total, histone acetylation increases gene transcriptional activity by relaxing chromatin (de Ruijter et al., 2003). The role of histone acetylation in memory has been intensively explored in recent years. Increasing histone H3 and H4 acetylation by means of HDAC inhibitors in the dorsal hippocampus enhances several types of hippocampal-dependent memories (Stefanko et al., 2009; Haettig et al., 2011), and also rescued hippocampal memory deficits in mouse models of AD (Ricobaraza et al., 2009; Kilgore et al., 2010) and on aged rats (Neidl et al., 2016). Different classes of HDAC can be distinctly involved in memory formation or in neurodegeneration. For instance, *Hdac1* expression is upregulated in a mouse model for Huntington’s disease (Quinti et al., 2010). However, mice lacking or overexpressing neuronal HDAC1 from early developmental stages are viable and display normal memory function (Guan et al., 2009). Here, a significant increase in H3 and H4 acetylation levels were found after EE in SAMP8. Although changes in *Hdac1. Sirt2*, and *Sirt6* revealed an increase in gene expression, *Hdac2* gene expression was significantly reduced, pointing to a major role of this deacetylase in the beneficial action of EE on the SAMP8 strain (Griñán-Ferré et al., 2015). Interestingly, HDAC2 is one of the major regulators involved in the regulation of long-term memory. Specifically, mice overexpressing HDAC2 exhibited memory impairment, while HDAC2-knockout mice demonstrated enhanced synaptic plasticity (Guan et al., 2009).

On the other hand, and in the same line of all previously mentioned herein, we found an increase in two transcriptional regulatory pathways; Nrf2 and NF-κB, despite the hypermethylation induced by EE. Thus, we can deduce that EE-induced hypermethylation of low-density CpG islands were the promoter for Nrf2, the Antioxidant Response Element (ARE), and for NF-κB.

It is well known that OS possesses a pre-eminent role in the pathogenesis of AD, and in addition, decreased expression of Nrf2 has been reported in AD (Cao et al., 2015). In response to OS, Nrf2 is translocated from the cytoplasm to the nucleus and subsequently binds with ARE, in turn promoting the expression of a variety of OS-related genes (Alam et al., 1999).

We found that in SAMP8 EE, there was significant Nrf2 nuclear translocation, and this in turn could induce the transcription of Heme oxygenase (decycling) 1 (*Hmox1*), Aldehyde oxidase 1 (*Aox1*), and Cyclooxygenase 2 (*Cox2*) genes. *Hmox1* can exert cytoprotective effects against OS, whereas *Aox1* and *Cox2* mediated Reactive Oxygen Species (ROS). These latter factors can act as hormetic regulators in an oxidative environment, favoring the cellular defenses confronting OS (Cai et al., 2012; Maeda et al., 2012). Other target genes, such as Aldehyde dehydrogenase 2 (*Aldh2*), were not affected (data not shown). It is also relevant that memory improvement in Nrf2-level modulation has been reported in aged APP/PS1 mice (Kanninen et al., 2009) and in memory-impaired rats (Dwivedi et al., 2013).

On the other hand, the high levels of inflammatory mediators can increase a physiological response to certain pathological conditions in the nervous system, as AD or stroke, and It has been suggested that modulation of the signaling pathways of these mediators could be a potential therapeutic approach (Buga et al., 2013; Carriba and Comella, 2015). Here, we have studied NF-κB, that is a transcription factor in positive feedback with and exercising a regulatory role in relationship to classic pro-inflammatory signals that also impacts cell survival and apoptosis, allowing cells to adapt and respond to environmental changes (Oeckinghaus and Ghosh, 2009). In a previous work, we described that EE induced a significant decrease of *IL-6* gene expression in the SAMP8 EE group compared with SAMP8 Ct (Griñán-Ferré et al., 2015). IL-6 is a pleiotropic inflammatory cytokine secreted by activated glia in the Central Nervous System (CNS) and is involved in mood disorders such as depression, also in aging process and the pathogenesis of neurodegenerative diseases such as AD (Quintanilla et al., 2004; Popa-Wagner et al., 2014). Our results demonstrate significant lowest genetic expression for *IL-6*, and also for *Cxcl10*, but not for Tumor necrosis factor alpha (*Tnf-α*) in the SAMP8 EE group. C-X-C motif chemokine 10 (*Cxcl10*) CXCL10 is secreted by several cell types in response to InterFeroN gamma (IFN-γ) and was found increased in AD mouse models (Zaheer et al., 2013). These results coincide with a decrease in the nuclear translocation of proinflammatory transcription factor NF-κB in SAMP8 mouse EE.

**FIGURE 6:**
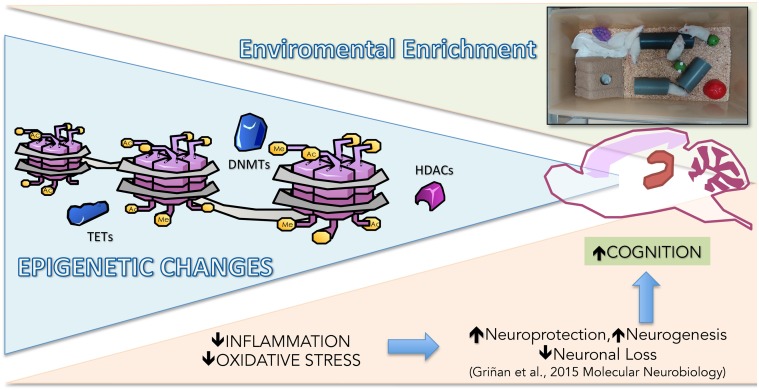
**Representative rendering of epigenetic and molecular mechanisms modified in Senescence-Accelerated Prone Mice P8 (SAMP8) after EE**.

Taken together, our results showed that crucial epigenetic modifications were demonstrated in the SAMP8 EE paradigm, reducing proinflammatory and OS pathways. Therefore, epigenetics orchestrated the homeostasis among DNA methylation, histone acetylation, and transcription factors, acting as a crosstalk mechanism and leading cognitive improvement and neuroprotection after EE in SAMP8 (**Figure 6**).

## Author Contributions

CG-F, DP-I, VP-A, DP-C, and JC-A performed the experiments. CG-F, MTR, and DP-C carried out the experimental intervention. CG-F and VP-A performed behavior experiments. DP-I and JC-A performed ELISA, and western blot analysis. CG-F and DP-I performed the RT-PCR experiments. CG-F, AC, and DO-S analyzed the data and drafted the manuscript. MP designed the experiments and supervised the study. All authors read and approved the final manuscript. All authors read and approved the final manuscript.

## Conflict of Interest Statement

The authors declare that the research was conducted in the absence of any commercial or financial relationships that could be construed as a potential conflict of interest.

## References

[B1] Alam J. Stewart D. Touchard C. Boinapally S. Choi A. M. Cook J. L. (1999). Nrf2, a Cap’n’Collar transcription factor, regulates induction of the heme oxygenase-1 gene. *J. Biol. Chem.* 274 26071–26078. 10.1074/jbc.274.37.26071 10473555

[B2] Alvarez-López M. J. Castro-Freire M. Cosín-Tomás M. Sanchez-Roige S. Lalanza J. F. Del Valle J (2013). Long-term exercise modulates hippocampal gene expression in senescent female mice. *J. Alzheimers Dis.* 33 1177–1190. 10.3233/JAD-121264 23168450

[B3] Avramopoulos D. (2009). Genetics of Alzheimer’s disease: recent advances. *Genome Med.* 1 34 10.1186/gm34 PMC2664945 19341505

[B4] Barak B. Shvarts-Serebro I. Modai S. Gilam A. Okun E. Michaelson D. M. (2013). Opposing actions of environmental enrichment and Alzheimer’s disease on the expression of hippocampal microRNAs in mouse models. *Transl. Psychiatry* 3 e304 10.1038/tp.2013.77 PMC3784766 24022509

[B5] Barrès R. Yan J. Egan B. Treebak J. T. Rasmussen M. Fritz T. (2012). Acute exercise remodels promoter methylation in human skeletal muscle. *Cell Metab.* 15 405–411. 10.1016/j.cmet.2012.01.001 22405075

[B6] Bradley-Whitman M. A. Lovell M. A. (2013). Epigenetic changes in the progression of Alzheimer’s disease. *Mech. Ageing Dev.* 134 486–495. 10.1016/j.mad.2013.08.005 24012631 PMC3857018

[B7] Buga A. M. Di Napoli M. Popa-Wagner A. (2013). Preclinical models of stroke in aged animals with or without comorbidities: role of neuroinflammation. *Biogerontology* 14 651–662. 10.1007/s10522-013-9465-0 24057280

[B8] Cai C. Teng L. Vu D. He J. Q. Guo Y. Li Q. (2012). The heme oxygenase 1 inducer (CoPP) protects human cardiac stem cells against apoptosis through activation of the extracellular signal-regulated kinase (ERK)/NRF2 signaling pathway and cytokine release. *J. Biol. Chem.* 287 33720–33732. 10.1074/jbc.M112.385542 22879597 PMC3460469

[B9] Canudas A. M. Gutierrez-Cuesta J. Rodríguez M. I. Acuña-Castroviejo D. Sureda F. X. Camins A. (2005). Hyperphosphorylation of microtubule-associated protein tau in senescence-accelerated mouse (SAM). *Mech. Ageing Dev.* 126 1300–1304. 10.1016/j.mad.2005.07.008 16171847

[B10] Cao H. Wang L. Chen B. Zheng P. He Y. Ding Y. (2015). DNA demethylation upregulated Nrf2 expression in Alzheimer’s disease cellular model. *Front. Aging Neurosci.* 7:244 10.3389/fnagi.2015.00244 PMC4700271 26779013

[B11] Carriba P. Comella J. X. (2015). Neurodegeneration and neuroinflammation: two processes, one target. *Neural Regen. Res.* 10 1581–1583. 10.4103/1673-5374.165208 26692848 PMC4660744

[B12] Cheng X. Zhou W. Zhang Y. (2014). The behavioral, pathological and therapeutic features of the senescence-accelerated mouse prone 8 strain as an Alzheimer’s disease animal model. *Ageing Res. Rev.* 13 13–37. 10.1016/j.arr.2013.10.002 24269312

[B13] Chestnut B. A. Chang Q. Price A. Lesuisse C. Wong M. Martin L. J. (2011). Epigenetic regulation of motor neuron cell death through DNA methylation. *J. Neurosci.* 31 16619–16636. 10.1523/JNEUROSCI.1639-11.2011 22090490 PMC3238138

[B14] Christensen B. C. Houseman E. A. Marsit C. J. Zheng S. Wrensch M. R. Wiemels J. L. (2009). Aging and environmental exposures alter tissue-specific DNA methylation dependent upon CpG island context. *PLoS Genet.* 5:e1000602 10.1371/journal.pgen.1000602 PMC2718614 19680444

[B15] Cosín-Tomás M. Alvarez-López M. J. Sanchez-Roige S. Lalanza J. F. Bayod S. Sanfeliu C. (2014). Epigenetic alterations in hippocampus of SAMP8 senescent mice and modulation by voluntary physical exercise. *Front. Aging Neurosci.* 6:51 10.3389/fnagi.2014.00051 PMC3960508 24688469

[B16] Costa D. A. Cracchiolo J. R. Bachstetter A. D. Hughes T. F. Bales K. R. Paul S. M. (2007). Enrichment improves cognition in AD mice by amyloid-related and unrelated mechanisms. *Neurobiol. Aging* 28 831–844. 10.1016/j.neurobiolaging.2006.04.009 16730391

[B17] Cristòfol R. Porquet D. Corpas R. Coto-Montes A. Serret J. Camins A. (2012). Neurons from senescence-accelerated SAMP8 mice are protected against frailty by the sirtuin 1 promoting agents melatonin and resveratrol. *J. Pineal Res.* 52 271–281. 10.1111/j.1600-079X.2011.00939.x 22085194

[B18] Day J. J. Sweatt J. D. (2010). DNA methylation and memory formation. *Nat. Neurosci.* 13 1319–1323. 10.1038/nn.2666 20975755 PMC3130618

[B19] de Ruijter A. J. M. van Gennip A. H. Caron H. N. Kemp S. van Kuilenburg A. B. P. (2003). Histone deacetylases (HDACs): characterization of the classical HDAC family. *Biochem. J.* 370(Pt 3), 737–749. 10.1042/BJ20021321 PMC1223209 12429021

[B20] Dwivedi S. Nagarajan R. Hanif K. Siddiqui H. H. Nath C. Shukla R. (2013). Standardized extract of bacopa monniera attenuates okadaic acid induced memory dysfunction in rats: effect on Nrf2 pathway. *Evid. Based Complement. Alternat. Med.* 2013:294501 10.1155/2013/294501 PMC3776558 24078822

[B21] Ennaceur A. Delacour J. (1988). A new one-trial test for neurobiological studies of memory in rats. 1. Behavioral data. *Behav. Brain Res.* 31 47–59. 10.1016/0166-4328(88)90157-X 3228475

[B22] Ennaceur A. Meliani K. (1992). A new one-trial test for neurobiological studies of memory in rats. III. Spatial vs. non-spatial working memory. *Behav. Brain Res.* 51 83–92. 10.1016/S0166-4328(05)80315-8 1482548

[B23] Fischer A. Sananbenesi F. Wang X. Dobbin M. Tsai L.-H. (2007). Recovery of learning and memory is associated with chromatin remodelling. *Nature* 447 178–182. 10.1038/nature05772 17468743

[B24] Fjell A. M. McEvoy L. Holland D. Dale A. M. Walhovd K. B. (2014). What is normal in normal aging? Effects of aging, amyloid and Alzheimer’s disease on the cerebral cortex and the hippocampus. *Prog. Neurobiol.* 117 20–40. 10.1016/j.pneurobio.2014.02.004 24548606 PMC4343307

[B25] Flax J. D. Soloway P. D. (2011). Methylation on the mind. *Nat. Neurosci.* 14 1494–1496. 10.1038/nn.2988 22119946

[B26] Gräff J. Tsai L.-H. (2013). Histone acetylation: molecular mnemonics on the chromatin. *Nat. Rev. Neurosci.* 14 97–111. 10.1038/nrn3427 23324667

[B27] Griñán-Ferré C. Pérez-Cáceres D. Gutiérrez-Zetina S. M. Camins A. Palomera-Avalos V. Ortuño-Sahagún D. (2015). Environmental enrichment improves behavior, cognition, and brain functional markers in young senescence-accelerated prone mice (SAMP8). *Mol. Neurobiol.* 53 2435–2450. 10.1007/s12035-015-9210-6 26014386

[B28] Griñán-Ferré C. Sarroca S. Ivanova A. Puigoriol-Illamola D. Aguado F. Camins A. (2016). Epigenetic mechanisms underlying cognitive impairment and Alzheimer disease hallmarks in 5XFAD mice. *Aging* 8 664–684. 10.18632/aging.100906 27013617 PMC4925821

[B29] Grupe A. Abraham R. Li Y. Rowland C. Hollingworth P. Morgan A. (2007). Evidence for novel susceptibility genes for late-onset Alzheimer’s disease from a genome-wide association study of putative functional variants. *Hum. Mol. Genet.* 16 865–873. 10.1093/hmg/ddm031 17317784

[B30] Guan J. S. Haggarty S. J. Giacometti E. Dannenberg J. H. Joseph N. Gao J. (2009). HDAC2 negatively regulates memory formation and synaptic plasticity. *Nature* 459 55–60. 10.1038/nature07925 19424149 PMC3498958

[B31] Haettig J. Stefanko D. P. Multani M. L. Figueroa D. X. McQuown S. C. Wood M. A. (2011). HDAC inhibition modulates hippocampus-dependent long-term memory for object location in a CBP-dependent manner. *Learn. Mem.* 18 71–79. 10.1101/lm.1986911 21224411 PMC3032579

[B32] Harold D. Abraham R. Hollingworth P. Sims R. Gerrish A. Hamshere M. L. (2009). Genome-wide association study identifies variants at CLU and PICALM associated with Alzheimer’s disease. *Nat. Genet.* 41 1088–1093. 10.1038/ng.440 19734902 PMC2845877

[B33] Hernandez D. G. Nalls M. A. Gibbs J. R. Arepalli S. van der Brug M Chong S. (2011). Distinct DNA methylation changes highly correlated with chronological age in the human brain. *Hum. Mol. Genet.* 20 1164–1172. 10.1093/hmg/ddq561 21216877 PMC3043665

[B34] Holliday R. (2006). Epigenetics: a historical overview. *Epigenetics* 1 76–80. 10.4161/epi.1.2.2762 17998809

[B35] Irier H. Street R. C. Dave R. Lin L. Cai C. Davis T. H. (2014). Environmental enrichment modulates 5-hydroxymethylcytosine dynamics in hippocampus. *Genomics* 104 376–382. 10.1016/j.ygeno.2014.08.019 25205305 PMC4252786

[B36] Ito S. Shen L. Dai Q. Wu S. C. Collins L. B. Swenberg J. A. (2011). Tet proteins can convert 5-methylcytosine to 5-formylcytosine and 5-carboxylcytosine. *Science* 333 1300–1303. 10.1126/science.1210597 21778364 PMC3495246

[B37] Jurgens H. A. Johnson R. W. (2012). Environmental enrichment attenuates hippocampal neuroinflammation and improves cognitive function during influenza infection. *Brain Behav. Immun.* 26 1006–1016. 10.1016/j.bbi.2012.05.015 22687335 PMC3454448

[B38] Kanninen K. Heikkinen R. Malm T. Rolova T. Kuhmonen S. Leinonen H. (2009). Intrahippocampal injection of a lentiviral vector expressing Nrf2 improves spatial learning in a mouse model of Alzheimer’s disease. *Proc. Natl. Acad. Sci. U.S.A.* 106 16505–16510. 10.1073/pnas.0908397106 19805328 PMC2752553

[B39] Kilgore M. Miller C. A. Fass D. M. Hennig K. M. Haggarty S. J. Sweatt J. D. (2010). Inhibitors of class 1 histone deacetylases reverse contextual memory deficits in a mouse model of Alzheimer’s disease. *Neuropsychopharmacology* 35 870–880. 10.1038/npp.2009.197 20010553 PMC3055373

[B40] Levenson J. M. O’Riordan K. J. Brown K. D. Trinh M. A. Molfese D. L. Sweatt J. D. (2004). Regulation of histone acetylation during memory formation in the hippocampus. *J. Biol. Chem.* 279 40545–40559. 10.1074/jbc.M402229200 15273246

[B41] Liu L. van Groen T. Kadish I. Tollefsbol T. O. (2009). DNA methylation impacts on learning and memory in aging. *Neurobiol. Aging* 30 549–560. 10.1016/j.neurobiolaging.2007.07.020 17850924 PMC2656583

[B42] López-Otín C. Blasco M. A. Partridge L. Serrano M. Kroemer G. (2013). The hallmarks of aging. *Cell* 153 1194–1217. 10.1016/j.cell.2013.05.039 23746838 PMC3836174

[B43] Madrigano J. Baccarelli A. Mittleman M. A. Wright R. O. Sparrow D. Vokonas P. S. (2011). Prolonged exposure to particulate pollution, genes associated with glutathione pathways, and DNA methylation in a cohort of older men. *Environ. Health Perspect.* 119 977–982. 10.1289/ehp.1002773 21385671 PMC3222977

[B44] Maeda K. Ohno T. Igarashi S. Yoshimura T. Yamashiro K. Sakai M. (2012). Aldehyde oxidase 1 gene is regulated by Nrf2 pathway. *Gene* 505 374–378. 10.1016/j.gene.2012.06.010 22705828

[B45] Marsit C. J. Christensen B. C. Houseman E. A. Karagas M. R. Wrensch M. R. Yeh R. F. (2009). Epigenetic profiling reveals etiologically distinct patterns of DNA methylation in head and neck squamous cell carcinoma. *Carcinogenesis* 30 416–422. 10.1093/carcin/bgp006 19126652 PMC2650795

[B46] Mastroeni D. Grover A. Delvaux E. Whiteside C. Coleman P. D. Rogers J. (2011). Epigenetic mechanisms in Alzheimer’s disease. *Neurobiol. Aging* 32 1161–1180. 10.1016/j.neurobiolaging.2010.08.017 21482442 PMC3115415

[B47] Mastroeni D. McKee A. Grover A. Rogers J. Coleman P. D. (2009). Epigenetic differences in cortical neurons from a pair of monozygotic twins discordant for Alzheimer’s disease. *PLoS ONE* 4:e6617 10.1371/journal.pone.0006617 PMC2719870 19672297

[B48] Mikaelsson M. A. Miller C. A. (2011). The path to epigenetic treatment of memory disorders. *Neurobiol. Learn. Mem.* 96 13–18. 10.1016/j.nlm.2011.02.003 21320618 PMC3217332

[B49] Millan M. J. (2014). The epigenetic dimension of Alzheimer’s disease: causal, consequence, or curiosity? *Dialog. Clin. Neurosci.* 16 373–393. 10.31887/DCNS.2014.16.3/mmillan PMC4214179 25364287

[B50] Miller C. A. Sweatt J. D. (2007). Covalent modification of DNA regulates memory formation. *Neuron* 53 857–869. 10.1016/j.neuron.2007.02.022 17359920

[B51] Morley J. E. Armbrecht H. J. Farr S. A. Kumar V. B. (2012a). The senescence accelerated mouse (SAMP8) as a model for oxidative stress and Alzheimer’s disease. *Biochim. Biophys. Acta* 1822 650–656. 10.1016/j.bbadis.2011.11.015 22142563

[B52] Morley J. E. Farr S. A. Kumar V. B. Armbrecht H. J. (2012b). The SAMP8 mouse: a model to develop therapeutic interventions for Alzheimer’s disease. *Curr. Pharm. Des.* 18 1123–1130. 10.2174/138161212799315795 22288401

[B53] Morris M. J. Adachi M. Na E. S. Monteggia L. M. (2014). Selective role for DNMT3a in learning and memory. *Neurobiol. Learn. Mem.* 115 30–37. 10.1016/j.nlm.2014.06.005 24937014 PMC4250315

[B54] Morris R. G. M. (1981). Spatial localization does not requiere the presence of local cues. *Learn. Motiv.* 12 239–260. 10.1016/0023-9690(81)90020-5

[B55] Neidl R. Schneider A. Bousiges O. Majchrzak M. Barbelivien A. de Vasconcelos A. P. (2016). Late-life environmental enrichment induces acetylation events and nuclear factor κB-dependent regulations in the hippocampus of aged rats showing improved plasticity and learning. *J. Neurosci.* 36 4351–4361. 10.1523/JNEUROSCI.3239-15.2016 27076430 PMC6601779

[B56] Oeckinghaus A. Ghosh S. (2009). The NF-kappaB family of transcription factors and its regulation. *Cold Spring Harb. Perspect. Biol.* 1:a000034 10.1101/cshperspect.a000034 PMC2773619 20066092

[B57] Pallàs M. (2012). Senescence-accelerated mice P8: a tool to study brain aging and Alzheimer’s disease in a mouse model. *ISRN Cell Biol.* 2012 1–12. 10.5402/2012/917167

[B58] Pallas M. Camins A. Smith M. A. Perry G. Lee H. Casadesus G. (2008). From aging to Alzheimer’s disease: unveiling “the switch” with the senescence-accelerated mouse model (SAMP8). *J. Alzheimers Dis.* 15 615–624. 19096160 10.3233/jad-2008-15408

[B59] Popa-Wagner A. Buga A. M. Adrian-Tica A. Valeria Albu C. (2014). Perfusion deficits, inflammation and aging precipitate depressive behaviour. *Biogerontology* 15 439–448. 10.1007/s10522-014.9516-1 25033986

[B60] Quintanilla R. A. Orellana D. I. González-Billault C. Maccioni R. B. (2004). Interleukin-6 induces Alzheimer-type phosphorylation of tau protein by deregulating the cdk5/p35 pathway. *Exp. Cell Res.* 295 245–257. 10.1016/j.yexcr.2004.01.002 15051507

[B61] Quinti L. Chopra V. Rotili D. Valente S. Amore A. Franci G. (2010). Evaluation of histone deacetylases as drug targets in Huntington’s disease models. Study of HDACs in brain tissues from R6/2 and CAG140 knock-in HD mouse models and human patients and in a neuronal HD cell model. *PLoS Curr.* 2:RRN1172 10.1371/currents.RRN1172 PMC2943247 20877454

[B62] Richardson B. (2003). Impact of aging on DNA methylation. *Ageing Res. Rev.* 2 245–261. 10.1016/S1568-1637(03)00010-2 12726774

[B63] Ricobaraza A. Cuadrado-Tejedor M. Pérez-Mediavilla A. Frechilla D. Del Río J. García-Osta A. (2009). Phenylbutyrate ameliorates cognitive deficit and reduces tau pathology in an Alzheimer’s disease mouse model. *Neuropsychopharmacology* 34 1721–1732. 10.1038/npp.2008.229 19145227

[B64] Snow W. M. Pahlavan P. S. Djordjevic J. McAllister D. Platt E. E. Alashmali S. (2015). Morris water maze training in mice elevates hippocampal levels of transcription factors nuclear factor (erythroid-derived 2)-like 2 and nuclear factor kappa B p65. *Front. Mol. Neurosci.* 8:70 10.3389/fnmol.2015.00070 PMC4649017 26635523

[B65] Spiegel A. M. Sewal A. S. Rapp P. R. (2014). Epigenetic contributions to cognitive aging: disentangling mindspan and lifespan. *Learn. Mem.* 21 569–574. 10.1101/lm.033506.113 25227252 PMC4175498

[B66] Stefanko D. P. Barrett R. M. Ly A. R. Reolon G. K. Wood M. A. (2009). Modulation of long-term memory for object recognition via HDAC inhibition. *Proc. Natl. Acad. Sci. U.S.A.* 106 9447–9452. 10.1073/pnas.0903964106 19470462 PMC2695069

[B67] Sureda F. X. Gutierrez-Cuesta J. Romeu M. Mulero M. Canudas A. M. Camins A. (2006). Changes in oxidative stress parameters and neurodegeneration markers in the brain of the senescence-accelerated mice SAMP-8. *Exp. Gerontol.* 41 360–367. 10.1016/j.exger.2006.01.015 16542809

[B68] Takeda T. (2009). Senescence-accelerated mouse (SAM) with special references to neurodegeneration models, SAMP8 and SAMP10 mice. *Neurochem. Res.* 34 639–659. 10.1007/s11064-009-9922-y 19199030

[B69] Weber M. Hellmann I. Stadler M. B. Ramos L. Pääbo S. Rebhan M. (2007). Distribution, silencing potential and evolutionary impact of promoter DNA methylation in the human genome. *Nat. Genet.* 39 457–466. 10.1038/ng1990 17334365

[B70] Williamson L. L. Chao A. Bilbo S. D. (2012). Environmental enrichment alters glial antigen expression and neuroimmune function in the adult rat hippocampus. *Brain Behav. Immun.* 26 500–510. 10.1016/j.bbi.2012.01.003 22281279 PMC3294275

[B71] Wu S. C. Zhang Y. (2010). Active DNA demethylation: many roads lead to Rome. *Nat. Rev. Mol. Cell Biol.* 11 607–620. 10.1038/nrm2950 20683471 PMC3711520

[B72] Yuan Z. Wang M. Yan B. Gu P. Jiang X. Yang X. (2012). An enriched environment improves cognitive performance in mice from the senescence-accelerated prone mouse 8 strain: role of upregulated neurotrophic factor expression in the hippocampus. *Neural Regen. Res.* 7 1797–1804. 10.3969/j.issn.1673-5374.2012.23.006 25624804 PMC4302529

[B73] Zaheer S. Thangavel R. Wu Y. Khan M. M. Kempuraj D. Zaheer A. (2013). Enhanced expression of glia maturation factor correlates with glial activation in the brain of triple transgenic Alzheimer’s disease mice. *Neurochem. Res.* 38 218–225. 10.1007/s11064-012-0913-z 23086473 PMC3527678

[B74] Zhao Z. Fan L. Fortress A. M. Boulware M. I. Frick K. M. (2012). Hippocampal histone acetylation regulates object recognition and the estradiol-induced enhancement of object recognition. *J. Neurosci.* 32 2344–2351. 10.1523/JNEUROSCI.5819-11.2012 22396409 PMC3401048

